# A systematic review of the concept and clinical applications of bone marrow aspirate concentrate in tendon pathology

**DOI:** 10.1051/sicotj/2017039

**Published:** 2017-10-09

**Authors:** Mohamed A. Imam, James Holton, Saman Horriat, Ahmed S. Negida, Florian Grubhofer, Rohit Gupta, Ali Narvani, Martyn Snow

**Affiliations:** 1 Department of Trauma and Orthopaedics, Faculty of Medicine, Suez Canal University Circular road Ismailia 41111 Egypt; 2 The Royal Orthopaedic Hospital Birmingham B31 2AP UK; 3 Birmingham University Birmingham B15 2TT UK; 4 St George Hospital London SW17 0QT UK; 5 Faculty of Medicine, Zagzig University 44519 Zagzig Egypt; 6 Department of Orthopaedics, Balgrist University Hospital, University of Zurich Forchstrasse 340 8008 Zürich Switzerland; 7 Ashford and St Peters Hospitals Chertsey KT16 0PZ UK; 8 Regenerative Medicine, Aston University, Aston Triangle Birmingham B4 7ET UK

**Keywords:** BMAC, Stem Cells, Bone marrow aspirate concentrate, Tendon, Tendinopathy

## Abstract

Tendon pathologies are a group of musculoskeletal conditions frequently seen in clinical practice. They can be broadly classified into traumatic, degenerative and overuse-related tendinopathies. Rotator cuff tears, Achilles tendinopathy and tennis elbow are common examples of these conditions. Conventional treatments have shown inconsistent outcomes and might fail to provide satisfactory clinical improvement. With the growing trend towards the use of mesenchymal stem cells (MSCs) in other branches of medicine, there is an increasing interest in treating tendon pathologies using the bone marrow MSC. In this article, we provide a systematic literature review documenting the current status of the use of bone marrow aspirate concentrate (BMAC) for the treatment of tendon pathologies. We also asked the question on the safety of BMAC and whether there are potential complications associated with BMAC therapy. Our hypothesis is that the use of BMAC provides safe clinical benefit when used for the treatment of tendinopathy or as a biological augmentation of tendon repair. We followed the Preferred Reporting Items for Systematic Reviews and Meta-Analyses (PRISMA) checklist while preparing this systematic review. A literature search was carried out including the online databases of PubMed, EMBASE, ClinicalTrial.gov and the Cochrane Library from 1960 to the end of May 2015. Relevant studies were selected and critically appraised. Data from eligible studies were extracted and classified per type of tendon pathology. We included 37 articles discussing the application and use of BMAC for the treatment of tendon pathologies. The Critical Appraisal Skills Program (CASP) appraisal confirmed a satisfactory standard of 37 studies. Studies were sub-categorised into: techniques of extraction, processing and microscopic examination of BMAC (*n* = 18), where five studies looked at the evaluation of aspiration techniques (*n* = 5), augmentation of rotator cuff tears (*n* = 5), augmentation of tendo-achilles tendon (*n* = 1), treatment of gluteal tendon injuries (*n* = 1), management of elbow epicondylitis (*n* = 2), management of patellar tendinopathy (*n* = 1) and complications related to BMAC (*n* = 5). Multiple experimental studies investigated the use of BMAC for tendon repair; nonetheless, there are only limited clinical studies available in this field. Unfortunately, due to the scarcity of studies, which were mainly case series, the current level of evidence is weak. We strongly recommend further future randomised controlled studies in this field to allow scientists and clinicians make evidence-based conclusions.

## Introduction

Tendon pathologies are a group of musculoskeletal conditions frequently seen in clinical practice. Tendons convey forces from muscles to bone and are repetitively exposed to mechanical loading. Per se, they are vulnerable to pathological transformations, known as tendinopathy. Tendinopathy is a comprehensive term including painful disorders ensuing in and around tendons. Their common clinical presentation is with pain and/or weakness and they can have a significant influence on a patient’s quality of life. Tendinopathy is particularly predominant in activities that include repetitive movements, indicative that the increased mechanical loading acting on tendons is a main contributor to the development of tendinopathy [1]. Yet, the exact pathogenesis of tendinopathy remains unclear. The characteristic histopathological characteristics of tendinopathy have been recognised, comprising the accumulation of fat cells, mucoid degeneration, tissue calcification or combinations of all [2]. These findings suggest that tendons contain cells with the potential to exhibit multi-phenotypes that differ from tenocytes, the resident cells in tendons, which express the fibroblast phenotype [3].

Conventional treatments, such as non-steroidal anti-inflammatory drugs (NSAIDs), corticosteroid injections, exercise-based physical therapy, physical therapy modalities, shock wave therapy, sclerotherapy, nitric oxide patches, surgery, and growth factors, have shown inconsistent outcomes. With the growing evidence advocating the use of mesenchymal stem cells (MSCs) in many branches of medicine, clinicians have shown considerable interest in treating tendon pathologies using the bone marrow MSC derived from Bone Marrow Aspirate (BMA). The key characteristic of bone marrow MSCs is their capability to proliferate and differentiate into a variety of cell lines and therefore instigate repair [4, 5]. MSC expansion for therapeutic use has recently been classified as an advanced therapeutic medicinal product, which results in significant expense and regulation [6]. Bone Marrow Aspirate Concentrate (BMAC) is generated in a one-step process and attempts to concentrate the mononucleate cells in order to potentially harness its reparative potential [7]. In vitro studies have shown promising results with the use of BMAC as an augment for Achilles tendon repair [8]. By 28 days after surgical repair, the failure load for rat Achilles treated with BMAC becomes equivalent to that of uninjured tendon [9].

In this article, we provide a systematic literature review documenting the current status of the use of BMAC for the treatment of tendon pathologies. We also assessed potential complications associated with the BMAC therapy. Our hypothesis is that the use of BMAC provides clinical benefit when used for the treatment of tendinopathy or as a biological augmentation of tendon repair.

## Methods

### Criteria for selecting studies for this review

We included studies that fulfill the following criteria:

1. Studies describing the development and techniques of BMAC.
2. Studies reporting the safety and/or efficacy of BMAC in tendon pathologies.
3. Studies on human subjects with tendon pathologies.

We excluded studies that were not on humans. There were no restrictions in terms of language and study design.

### Search strategy

We performed a computer literature search of online databases: PubMed, EMBASE, ClinicalTrial.gov and the Cochrane Library from 1960 to the end of August 2016. We used the following keywords: (“Bone” AND “Marrow” AND “Aspirate” AND “Concentrate”).

### Study identification

The title and abstract of each study within the results list were reviewed independently by three authors (MAI, JH, and SH). Any disagreement was resolved by discussion with the senior author. Full-text papers of relevant studies were subsequently obtained and reviewed against the eligibility criteria. Then, full texts of the eligible studies were further evaluated and references were checked for more suitable studies. The included studies were classified according to the type of tendon pathology.

### Critical appraisal

Two authors using the Critical Appraisal Skills Program (CASP) checklist [10] independently appraised eligible studies. For the narrative review, relevant studies were included, irrespective of the methodology or level of evidence.

## Results and discussion

**Figure 1.**
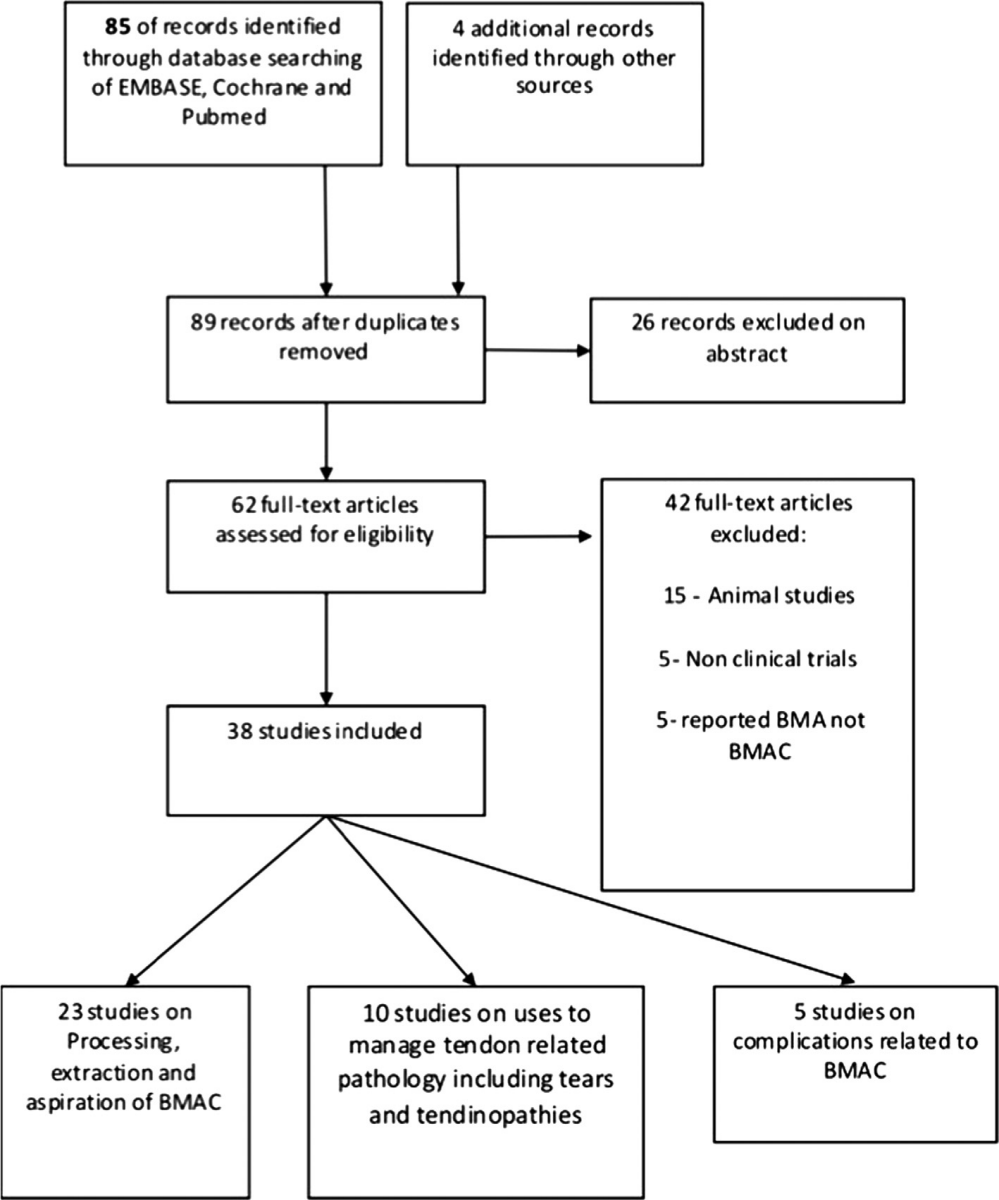
Flow diagram of the search results.

The literature search yielded 89 records. Of these, 37 were eligible for inclusion and assessment in this review (Figure 1).

The CASP appraisal confirmed a satisfactory standard of 37 studies. They all had clearly defined objectives, were well designed and conducted appropriately to meet them.

The published studies reported techniques of collection and preparation of BMAC in addition to its applications in many orthopaedic subspecialities. Studies could be sub-categorised into: techniques of extraction, processing and microscopic examination of BMAC (*n* = 18), where five studies looked at the evaluation of aspiration techniques (*n* = 5), augmentation of rotator cuff tears (*n* = 5), augmentation of Tendo-Achilles tendon (*n* = 1), treatment of gluteal tendon injuries (*n* = 1), management of elbow epicondylitis (*n* = 2), management of patellar tendinopathy (*n* = 1) and complications related to BMAC (*n* = 5).

### Bone marrow composition

The cellular composition of the marrow aspirate in normal individuals has been evaluated by Bain [11]. They assessed small volumes (0.1–0.2 mL) in 50 subjects. They reported that neutrophils and erythroblasts formed a major percentage of the aspirate with differences noticed between the sexes. Nevertheless, erythroblasts were higher at 28.1% in men compared to 22.5% in women, tending to represent the variations in adult haemoglobin concentrations. The other components included lymphocytes 13.1%, eosinophils 2.2%, blast cells 1.4, monocytes 1.3%, and basophils 0.1%. From this analysis it was notable that there was a broad variation in the megakaryocyte percentage. Subsequently, this explains the extensive variation in normal ranges in the adult population [11]. Additionally, Yamamura et al. [12] gauged the configuration of the BMA on the cellular level by laser photometry. Their observations on the average subpopulation percentages per 0.5 mL of BMA were consistent with the findings of Bain [11]. They observed that neutrophils constitute the largest cell population (45.9%) in marrow aspirate, followed by erythroblasts (24.6%), lymphocytes (13.4%), monocytes (8.1%), eosinophils (2.7%) and basophils (0.1%) [7]. Blast cells made up 2.1% of the cell populations analysed [7]. Although the mechanism of cellular analysis differs, the percentage of each cell type is largely similar. Kim et al. [13] found similar percentages using a flow cytometry technique analysing 2 mL of BMA from haematology patients without active disease.

### Bone marrow harvest

Different marrow harvest sources have been described in the literature. In a study carried out by Hyer et al. [14], they compared the iliac crest, tibia and calcaneus. These are regarded as the commonly available areas for harvesting the BMA. They assessed the number of osteoblastic connective tissue progenitor cells (OPG) from the aspirates harvested. They demonstrated that the iliac crest produced a superior average concentration of OPG compared to the tibia and calcaneus. Consequently, a larger amount of BMA is required when aspirating from the tibia or calcaneus to achieve a similar aspirate of MSCs gained from the iliac crest. Furthermore, Pierini et al. [15] inspected the harvest achieved from the iliac crest and demonstrated that the posterior iliac crest produced 1.6-fold more OPG than the anterior crest. Nevertheless, the posterior iliac crest is more technically challenging during the surgical procedures than the anterior crest. Variables like gender, smoking status and diabetes were not predictive of osteoblastic progenitor cell concentration [14].

Muschler et al. [16] showed that as the quantity of marrow aspirate rose from 2 to 4 mL, the number of OPG cells shrank by 50%. These observations were originally reported on the BMA from the iliac crest. Interestingly, this finding is not exclusive to the iliac crests, as it was also shown in other sources for BMA including the spine. This is likely due to the proportion of blood in the subsequent samples diluting the concentration of progenitor cells and colony forming units/stem cells.

Aiming at achieving safe aspiration of the marrow, Hernigou et al. [17] have developed the sector rule for BMA from the iliac crest, which he popularised as the concept of “safety zones”. They studied the anatomy of the iliac bone including the adjacent neurovascular elements that are susceptible to damage by the trocar when it is introduced into the iliac crest. The authors used computed tomography (CT) to examine 48 iliac crests in 24 pelvises. They split the crest into six even sectors in an anteroposterior course. They studied 480 trocar entry points carried out by six surgeons in 120 subjects. They established that the sector system reliably envisaged safe and unsafe zones for placing the trocar in the iliac crest. They perceived a larger risk of breaches of the cortical bone in obese patients, and this risk was reduced in the hands of more experienced surgeons. Ninety-four breaches out of 480 entry points happened with increased risks observed in the thinner sectors of the iliac crest. Furthermore, there is added risk of wounding the external iliac artery in the four most anterior sectors particularly in females. Instead, posterior sectors were associated with increased risk of sciatic nerve and gluteal vessel injury when the trocar was inserted more than 6 cm into the posterior iliac crest. They established that the sector rule is a reliable system to use for aspiration of the bone marrow.

Hernigou et al. [18] in another study recognised that the usage of 10 mL syringes to harvest BMA was superior to that of 50 mL syringes. In 30 patients the 10 mL syringe aspirations resulted in progenitor cell concentrations on average 300% higher. This was believed to be secondary to a larger negative pressure generated in the 10 mL syringe, which preferentially removed bone marrow cells and reduced blood contamination.

### Bone marrow concentration

The main concern in using BMA to stimulate tissue repair/regeneration is the low concentration of MSCs found within it, as only 0.001% of nucleated cells within BMA are actual MSCs. The reported concentration of MSCs in bone marrow is 7–30 cells per million nucleated cells [19]. To address this issue, various protocols have been developed to concentrate the nucleated cell numbers to produce BMAC. Therefore, increasing the amount of MSCs is an attempt to provide an effective environment for healing and regeneration [5, 7, 19]. Hernigou et al. [20] highlighted that the efficacy of BMAC is dependent on the amount of progenitor cells in the concentrate. They compared the quantity and concentration of these cells in both BMA and BMAC aspirated from the iliac crest when used for the treatment of atrophic non-union of the tibia. The BMA contained a mean of 612 ± 134 compared to 2579 ± 1121 cells per cm^3^ in the BMAC group. Reduced concentration of progenitor cells at the non-union sites was significantly associated (*P* < 0.01) with non-union [20].

Different techniques have been proposed to concentrate the bone marrow aspirate to form BMAC. These include the use of Ficoll density gradients in the laboratory setting and automated, closed centrifugation systems in the clinical setting [6]. Although these techniques increase the number of MSCs, they do not significantly increase the ratio of stem cells to other nucleated cells [5, 19, 21]. Centrifugation is the present technique of choice for the various commercially available products used in the clinical setting, although as shown in relation to platelet-rich plasma (PRP) there is a significant variation in the final end products achieved. Fortier et al. [22] compared the constituents of PRP and BMAC, and observed that there are reduced platelets and raised white blood cells (WBCs) in BMAC demonstrating that this is a very different substance to PRP with a likely different mechanism of action.

The concept of BMAC is to improve the recovery of the nucleated cells from the marrow aspirate, while decreasing the recovery of non-nucleated cells such as Red blood cells (RBCs). The exact mechanism of action of BMAC is currently not fully understood. Potentially the MSCs contained within the BMAC provide a direct cell source for repair of the host tissue. In addition, the nucleated cells may deliver various cytokines and growth factors into the delivery site to orchestrate and direct host repair [23–26]. Multiple reports have described clinical improvement in different conditions including stroke, diabetes, and Parkinson’s disease after the systemic or local use of bone marrow MSC [27, 28]. Until recently it was widely believed that after administration of bone marrow MSC, mesenchymal cells differentiate into the target cells and participate in the repair process. Nonetheless, it is suggested that healing capabilities of MSC are mainly through chemical mediators and cytokines which can communicate to the injured tissue and control cell proliferation, angiogenesis and inflammatory process required for repair [29]. Unfortunately, there are very limited published studies on the uses of BMAC in the management of tendon pathologies.

MSCs are believed to have multipotent plasticity with the capability to differentiate along multiple cell lineages such as cartilage, bone, tendon, muscle and nerve [4, 29–33]. Such multipotency has the potential to play an important role in the repair and reconstruction of multiple tissues across a number of orthopaedic specialties [7]. Various animal and investigational studies have shown that BMAC can influence tendon repair; still, there are few clinical studies available [34–36]. McCarrel and Fortier demonstrated potentially optimal release of growth factors for the management of injuries in tendons and ligaments [26]. In one equine study, BMAC harvested from the sternum improved the function of 72% horses with tendon/ligament pathology and allowed them to return back to race competition [37]. Similarly, in another case series on racehorses with tendon and ligament injuries treated with BMAC and PRP, authors reported significant and marked improvement in all horses and a return to competition in 84% [38].

### Rotator cuff tears

Tendon pathologies can range from tendinopathy to complete tear, with the rotator cuff, Achilles and the common extensor origin the most commonly affected. Despite improvements in surgical knowledge and technology, repairs of full-thickness cuff tears may be associated with re-tear rates of 25%–57% [39, 40]. Nowadays, a better knowledge about the healing of rotator cuff tendons and the nature of tears has guided the pursuit for an answer that tackles not only the mechanical aspects but also the biological side of healing. Such a resolution may lead to better healing and subsequent regeneration of the tendons. In a case series reported by Ellera Gomes et al. [41], 14 patients (nine women, five men) with full-thickness rotator cuff tears were managed with trans-osseous stitches augmented with BMAC utilizing a mini-open technique. All patients were followed up for a minimum of one year. Magnetic resonance imaging (MRI) scans were undertaken to assess tendon integrity at one-year post-surgery. They reported significant improvement in the University of California Los Angeles (UCLA) score from 12 ± 3.0 to 31 ± 3.2 at 12 months post-surgery. Tendon integrity was maintained in all cases while six patients (42%) displayed the formation of a high-signal intensity at the critical zone. One revision was reported at two years follow-up. The authors concluded that BMAC could be safely implanted as an augment in rotator cuff repairs.

Hernigou et al. [42] evaluated the efficiency of BMAC in augmenting arthroscopic single row rotator cuff repair in 45 patients. Their primary objective was to compare tendon healing in the BMAC group to that of a matched control group of 45 patients without augmentation with BMAC. They found that patients treated with BMAC demonstrated superior outcomes in terms of tendon healing rate and enhanced quality of the repaired tendon, as demonstrated by ultrasound and MRI. Forty-five shoulders (100%) in the BMAC group demonstrated tendon healing by six months, compared to only 30 shoulders (67%) in the control group at the same time point. Furthermore, at the latest follow-up of 10 years, the integrity of the repair was maintained in 39 (87%) shoulders in the BMAC group compared to only 20 (44%) shoulders in the control group.

In 2015, Havlas et al. [43] reported the efficiency of augmenting cuff repairs with BMAC. BMAC was utilised in combination with single row rotator cuff repair in 45 patients. They compared the outcomes to those of a control group of 45 patients in which BMAC was not utilised. They reported that the BMAC injection enhanced the healing rate and improved the quality of the repaired tendon as determined by the MRI and Ultrasound (US). Forty-five (100%) compared to 30 (67%) rotator cuff tears had healed by six months as shown on MRI in the BMAC and control group, respectively. The BMAC injection also prevented further ruptures during the next 10 years. This study showed that a significant improvement in healing outcomes could be achieved using BMAC as an adjunct to rotator cuff repair. The authors reported no adverse effects of BMAC therapy post-surgery.

Centeno et al. [44] in 2015 reported, in a prospective multicentre study, the use of BMAC for the treatment of osteoarthritis (OA) with and without rotator cuff pathology. A total of 115 shoulders were treated with BMAC injection principally for glenohumeral OA with or without rotator cuff tear. The mean disabilities of the arm, shoulder and hand score (DASH) and Visual Analogue Score (VAS) improved significantly from 36.1 to 17.1 (*P* < 0.001) and 4.3 to 2.4 (*P* < 0.001), respectively. These results were consistent with a mean subjective improvement of 48.8%. They reported no significant adverse events. These studies are summarised in Table 1.

**Table 1.** Summary of studies assessing the efficiency of BMAC in the management of rotator cuff tears.

Study ID	Design	Population	Intervention	Comparator	Outcome measure	Findings
Ellera Gomes et al. [41]	Single arm prospective study	Fourteen patients (nine women, five men) with full thickness rotator cuff tears	Trans-osseous stitches augmented with BMAC utilizing a mini-open technique	No control	University of California Los Angeles	From 12 ± 3.0 to 31 ± 3.2
					Tendon integrity (12 months)	Tendon integrity was maintained in 100% of cases.
Hernigou et al. [20]	Prospective, matched-control, study	Fifty-four patients	BMAC in augmenting arthroscopic single row rotator cuff repair	Matched control group of 45 patients without augmentation with BMAC	Tendon integrity (six months)	100% vs. 67%
					Tendon integrity (10 years)	87% vs. 44%
Centeno et al. [44]	Prospective multicentre cohort study	Patients with osteoarthritis with and without rotator cuff pathology	BMAC injection for the gleno-humeral OA	None	The arm, shoulder and hand score Visual analogue score Mean subjective improvement	Improved from 36.1 to 17.1 (*P* < 0.001) Improved from 4.3 to 2.4 (*P* < 0.001) 48.8%
Havlas et al. [43]	Prospective, matched-control, study	Forty-five patients	BMAC in augmenting arthroscopic single row rotator cuff repair	Matched control group of 45 patients without augmentation with BMAC	Tendon integrity (6 months)	(100%) compared to 30 (67%)

### Lower limb tendon injury

Recently, Stein et al. [45] reviewed 27 patients (28 tendons) with a mean age of 38.3 years with sport-related Achilles tendon rupture treated with combined open tendon repair and injection of BMAC. Their outcomes included complications, restoration of strength, range of motion, re-rupture, calf circumference, and the Achilles tendon Total Rupture Score (ATRS). They reported no re-rupture at final follow-up at an average of 30 months. They showed that 92% of patients resumed their sporting activity at six months. This cohort of patients attained a mean ATRS of 91 points at final follow-up. One soft tissue complication limited to superficial wound dehiscence was the reported significant complication, with no deep or superficial infection and no venous thromboembolic event. This study demonstrated excellent results, including no re-ruptures and early mobilization was observed in this cohort of patients. The efficacy of BMAC in the operative repair of acute Achilles tendon ruptures warrants further study.

Campbell et al. [46] reported the successful use of BMAC in symptomatic deficiency of the hip capsule and gluteus minimus tendon in a professional athlete after the removal of heterotopic ossification. Over 16 weeks, he received in total three PRP and two BMAC injections. Repeated BMAC injections improved his pain and strength as well as the integrity of the hip capsule and gluteus minimus tendon on MRI. The motion analysis results revealed enhancement in kicking performance while his subjective pain score steadily reduced over the treatment time period [46].

### Epicondylitis

Traditionally, the chief lesion in lateral epicondylitis was considered to be inflammatory granulation tissue in the tendinous portion of the common extensor origin. However, recent studies have failed to demonstrate the presence of the inflammatory cascade. Given the presence of tendon degeneration within this lesion the expression, epicondylosis, or tendinosis has been advocated as a more suitable term than epicondylitis. Tendon ruptures are dissimilar to bone, in that tendon healing does not result in complete healing and a return to the uninjured state. Conversely, a fibrovascular tissue is produced which yields to a tendon mechanically weaker than the native tendon [47].

Moon et al. [48] combined surgical intervention with BMAC to treat medial or lateral epicondylitis in 26 elbows (24 patients) that had failed non-operative treatment. They concluded that an injection of BMAC after arthroscopic debridement in elbow tendinitis established faster recovery of daily activities and clear improvement. They reported 100% improvement in their visual analogue pain scores and Mayo elbow performance scores (*P* < 0.001) at the six months time point. Ultrasound (US) examination of the treated elbow at the final postoperative follow-up revealed healthy uniform orientation of the tendon. In the cytokine analysis, significantly high levels of Interleukin-12 (IL-12), Interferon gamma-induced Protein 10 (IP-10), and Regulated on Activation, Normal T Cell Expressed and Secreted (RANTES) were detected.

Similar results were reported by Singh et al. [49], a total of 30 patients of formerly untreated lateral epicondylitis were administered a local injection of BMAC. They assessed the clinical outcome at baseline, two weeks, six weeks and 12 weeks using the Patient-Rated Tennis Elbow Evaluation (PRTEE) score. The baseline pre-injection mean PRTEE score was 72.8 ± 6.97, which decreased to a mean of 40.93 ± 5.94 at two weeks after injection, which was highly significant (*P* < 0.0001). In their final follow-up at 12 weeks, there was a significant improvement in the mean PRTEE score compared to the baseline score (72.8 ± 6.9 down to 14.86 ± 3.48). The authors concluded that an injection of growth factors and stem cells in the buffy coat layer of BMAC was an effective way of managing chronic lateral epicondylitis.

### Patella tendon tendinopathy

Pascual-Garrido et al. [50] reported the clinical results of a case series of ultrasound-guided injection of BMAC for refractory patella tendinitis unresponsive to non-operative treatment. Their report included eight patients (four males and four females) aged between 14 and 35 years. The study reported a significant improvement in pain and activity of daily living scores, knee-related quality of life and functional knee scores (International Knee Documentation Committee (IKDC) and Knee Injury and Osteoarthritis Outcome Score (KOOS)), in seven out of eight patients treated with ultrasound-guided injection of BMAC. Seven out of eight patients stated they would have the procedure again and categorised the outcome of their treatment as excellent. In this study, the number of nucleated cells in the BMA was 37 × 10^3^ and after concentration it reached to 45 × 10^3^.

### Potential risks

Marrow aspiration is thought to be a relatively safe procedure. As with all invasive procedures, patients should have consented with regard to all possible risks [51–54]. These risks can be associated with harvesting the BMA or with the application of BMAC.

#### Risks of harvest

In order to investigate the risks of BMA harvest, Bain [51] undertook a multicentre study surveying 19,259 procedures. Haemorrhage occurred in 11 (0.0005%) patients. Associated anticoagulation therapy was the most significant risk factor for haemorrhage. Superficial infection has been reported in two subjects, and both of them were successfully treated with antibiotics. While pain is dependent on the site of harvest, chronic pain can be a potential concern. The pain might not spontaneously resolve and could be managed by neuropathic pain medications [52, 54]. Neurovascular injury is another possible risk; the shared worry is the risk of injury to the neurovascular structures close to the harvest site. Injury to these structures may result in haemorrhage or problems with chronic pain. Moreover, pathological fractures are potential concern especially when there is an underlying metabolic problem like osteomalacia or bone density pathology like osteoporosis [51–54]. Lastly, death is an extreme adverse event that has been reported in one patient in 2001, due to the formation of a retroperitoneal haematoma after an aspirate from the posterior iliac crest [51].

#### Risks of administration

The relevant administration risks are dependent on what procedure is being carried out, but generally include the risks given below.

##### Infection

Infection is still a possibility at the administration site, although prophylactic antibiotics for the original procedure itself are common practice in many centers. This risk will be dependent on the procedure but routine universal aseptic precautions should be used in order to minimize the risk [51].

##### Fat embolism and respiratory complications

When used intraosseously, due to bone being permeable to liquefied material, fat embolisation is a potential risk [55]. Animal studies have shown fat particles in the lungs of dogs at post-mortem, nevertheless in human trials, adverse clinical outcomes in the form of respiratory complications or decreased oxygen saturation have not been reported [56]. Those subjects at a greater risk of embolization such as those with cardiac shunts should be considered as to their suitability to receive intraosseous BMAC. In all cases of intraosseous administration, patients should be monitored for the clinical signs of fat embolism [24]. In the clinical experience of Hernigou et al., no respiratory complications or changes in oxygen saturation have been seen [19]. Hitherto, considering Orlowski et al.’s findings, a BMAC intraosseous injection should be used with caution and should not be used in subjects with a right to left intracardiac shunt due to the risk of embolism to the vital organs.

Finally, there are limitations in this study. As for all systematic reviews, this is a pooled analysis of case series and retrospective studies of Level IV of evidence. Hence, there is the risk of bias and other confounding variables. Nevertheless, to the best of our knowledge, this is the first review examining the uses of BMAC in the management of tendon pathology. Moreover, there are so few studies to review that assess multiple different tendinopathies, which may respond differently to this treatment.

##### Risk of tumorigenesis

One of the greatest concerns with delivering BMAC into a new site is the possibility for tumorigenesis. Nevertheless, this theory is unproven in one big series by Hernigou et al. [57]. Between 1990 and 2006, they retrospectively reviewed 1873 patients managed with BMAC at a mean follow-up of 12.5 years. Patients were examined for the occurrence of cancer from the date of the first operation until death. They reviewed more than 7300 MRIs and 52,000 radiographs. They reported the diagnosis of malignancy in 53 cases. All 53 cancers were diagnosed in areas other than the BMAC injection site. Nonetheless, as per the reported incidence of cancer in the general population, the anticipated incidence over the same study period was theoretically between 97 and 108. This study found no increased cancer risk in patients after the application of BMAC either at the administration site or elsewhere in 1873 patients after an average follow-up period of 12.5 years.

## Conclusions

In conclusion, MSCs in BMAC are able to self-renew and possibly differentiate into different tissues. Furthermore, MSCs are known to control the immune system and have a conceivable positive paracrine influence. BMAC has been mostly used to encourage bone formation and treat avascular necrosis (AVN) of the femoral head, with encouraging results. There is increasing interest in the use of BMAC in tendon pathologies with early results displaying promise. Alongside well-designed randomised controlled clinical trials, further basic science work is required to investigate the therapeutic action of BMAC. The commercial processing of BMAC should be enhanced to accomplish a reliable end product, which will deliver predictable results.

We have investigated the clinical use of BMAC in tendon pathologies using all available published studies. Unfortunately, there is still limited evidence available given the small number of published studies. The reviewed articles did not report any significant complications directly related to the use of BMAC in tendon pathologies; however apart from one study with a 10-year follow-up [42], all other clinical studies had short- to medium-term follow-up. We believe there is sufficient promising data to warrant further well-designed clinical studies to allow scientists and clinicians to make evidence-based decisions regarding the use of BMAC in tendon injuries.

## Conflict of interest

The authors declare that they have no conflict of interest in relation with this paper.
